# Evolutionary and Functional Features of Copy Number Variation in the Cattle Genome

**DOI:** 10.3389/fgene.2016.00207

**Published:** 2016-11-22

**Authors:** Brittney N. Keel, Amanda K. Lindholm-Perry, Warren M. Snelling

**Affiliations:** Agricultural Research Service (USDA), Meat Animal Research CenterClay Center, NE, USA

**Keywords:** copy number variation, cattle genome, next-generation sequencing, deletion, duplication, gene expression, network centrality, selective constraint

## Abstract

Genomic structural variations are an important source of genetic diversity. Copy number variations (CNVs), gains and losses of large regions of genomic sequence between individuals of a species, have been associated with a wide variety of phenotypic traits. However, in cattle, as well as many other species, relatively little is understood about CNV, including frequency of CNVs in the genome, sizes, and locations, chromosomal properties, and evolutionary processes acting to shape CNV. In this work, we focused on copy number variation in the bovine genome, with the aim to detect CNVs in *Bos taurus* coding sequence and explore potential evolutionary mechanisms shaping these CNV. We identified and characterized CNV regions by utilizing exome sequence from 175 influential sires used in the Germplasm Evaluation project, representing 10 breeds. We examined various evolutionary and functional aspects of these CNVs, including selective constraint on CNV-overlapped genes, centrality of CNV genes in protein-protein interaction networks, and tissue-specific expression of CNV genes. Patterns of CNV in the *Bos taurus* genome reveal that reduced functional constraint and mutational bias may play a prominent role in shaping this type of structural variation.

## Introduction

Copy number variations (CNVs) are gains and losses of large regions of genomic sequence between individuals of a species (Mills et al., 2011). CNVs have been well-studied and linked to various phenotypic traits and diseases in humans and rodents (Cook and Scherer, 2008; Almal and Padh, 2012; Girirajan et al., 2013). Initial CNV studies have been performed in a number of domesticated animals: dog (Nicholas et al., 2011; Alvarez and Akey, 2012; Berglund et al., 2012), sheep (Fontanesi et al., 2011; Liu et al., 2013), pig (Fadista et al., 2008; Ramayo-Caldas et al., 2010; Chen et al., 2012; Paudel et al., 2013, 2015), chicken (Crooijmans et al., 2013; Yi et al., 2014), and goat (Fontanesi et al., 2010). These studies have linked many phenotypic traits to CNV, including chicken pea-comb phenotype (Wright et al., 2009) and white coat color in pigs and sheep (Johansson Moller et al., 1996; Norris and Whan, 2008).

Several studies have investigated CNV in the bovine genome. Cattle CNVs have been reported using a variety of platforms, including comparative genomic hybridization arrays (Liu et al., 2008, 2010; Fadista et al., 2010), the Illumina BovineHD BeadChip (Hou et al., 2012a; Wu et al., 2015; Aguilar et al., 2016; Prinsen et al., 2016; Xu et al., 2016), the Illumina BovineSNP50 BeadChip (Matukumalli et al., 2009; Bae et al., 2010; Hou et al., 2011, 2012b; Jiang et al., 2012; Bagnato et al., 2015; Ben Sassi et al., 2016), and next-generation sequencing (NGS) (Stothard et al., 2011; Zhan et al., 2011; Bickhart et al., 2012; Choi et al., 2013; Keel et al., 2016; Ben Sassi et al., 2016). In these studies, it is reported that copy number variable regions comprise ~2–7% of the cattle genome.

In cattle, as well as many other species, relatively little is known about the properties and dynamics of CNVs. Open questions remain about the frequency of CNVs in the genome, sizes, and locations, and chromosomal properties. In addition, the extent to which CNV affect phenotype is not well understood. In humans, it has been observed that two unrelated, healthy individuals can differ from one another in gene copy number across their genomes (Sabat et al., 2004), which raises uncertainty about the existence of a characteristic number of copies of any one gene. Of all of the topics related to CNVs, our knowledge of the functional and evolutionary impact of CNVs is the most limited.

Whole genome sequence (WGS) is often used in CNV discovery. However, until sequencing costs drop dramatically, it is simply not feasible to generate the high coverage (> 10x) whole genome sequence, suggested for CNV detection, on large numbers of animals. Due to its cost-effectiveness, WES is routinely used for the detection of coding sequence variation (Guo et al., 2013). In humans, the exome comprises approximately 1–3% of the genome, but accounts for over 85% of all mutations identified in Mendelian disorders (Ng et al., 2010), making it a desirable and practical approach for investigating variations in coding sequence.

In this study, we investigated some evolutionary and functional aspects of coding sequence copy number variation in the bovine genome. We first characterized CNV regions detected in whole exome sequence from 175 influential sires used in the USMARC Germplasm Evaluation project and identified genes overlapping with CNVRs. We then examined selective constraint on CNV genes to test the hypothesis that genes affected by CNV are subject to accelerated sequence evolution compared to copy number neutral genes. In addition, we utilized gene expression data and protein-protein interaction networks to investigate network centrality and tissue-specific expression patterns of CNV genes.

## Materials and methods

The DNA samples sequenced for this study were extracted from semen collected by commercial AI services and from blood archived under standard operating procedures for the U.S. Meat Animal Research Center tissue repository. The research did not involve experimentation on animals requiring IACUC approval.

### Sequencing and data acquisition of GPE sires

CNV were detected from whole exome sequence of 175 bulls used in Cycle VII of the USMARC germplasm evaluation (GPE) project. This included 122 purebred AI sires representing 10 different breeds, and 53 F_1_ natural service sires representing 10 different crosses of 7 breeds (Table 1). Bulls were selected for sequencing according to their influence on the GPE project (see Snelling et al., 2015 for full details). Exome sequence is available for download from the National Center for Biotechnology Information Sequence Read Archive (SRA) with Accession Number SRP076471.

**Table 1:** **Breeds of sequenced bulls from the USMARC germplasm evaluation (GPE) population used in this study**.

**Breed**	**Number of bulls**
Hereford	17
Angus	19
Simmental	16
Limousin	15
Charolais	17
Gelbvieh (German Yellow)	17
Red Angus	15
Shorthorn	3
Braunvieh (Brown Swiss)	1
Brahman	2
Charolais × Angus	2
Gelbvieh × Hereford	6
Simmental × Hereford	3
Simmental × Angus	4
Hereford × Angus	13
Limousin × Hereford	5
Gelbvieh × Angus	6
Red Angus × Hereford	7
Charolais × Hereford	3
Limousin × Angus	4

Exome sequencing was previously described by Snelling et al. (2015). Briefly, genomic DNA was extracted from semen and blood using standard DNA extraction protocols (phenol-choloroform extraction for semen and QIAamp DNA Mini Kit for blood), and sheared to an average size of 300 bp. Indexing adapters were added to allow identification of individual DNA samples from pools of 8 samples. The Agilent SureSelect Target Enrichment System Kit I and Kit II (Agilent Technologies, Inc., Santa Clara, CA) were used to generate a DNA library for each sample. Equal quantities of each indexed DNA library were pooled into groups of 8 for exome capture using the Agilent SureSelect XT Bovine capture reagent (Agilent Technologies, Inc., Santa Clara, CA). Exome capture libraries were then sequenced with the Illumina MiSeq technology (MiSeq Reagent Kit V2 and V3 chemistry; Illumina, San Diego, CA) to obtain a mean 20x coverage of targeted intervals.

Processing of the FASTQ files was done using the best practices established for the Genome Analysis Toolkit (GATK, Van der Auwera et al., 2013). Reads were removed if overall quality score was less than 20, if they contained more than three uncalled bases, or if they failed the Illumina chastity filter. TrimmomaticPE (Bolger et al., 2014) was used to trim Illumina adaptor sequences and low quality bases from the reads. The bowtie2 (Langmead and Salzberg, 2012) program was then used to map the reads to the UMD 3.1 genome assembly (Zimin et al., 2009).

### CNV detection and defining CNVRs

The cn.MOPS algorithm (Klambauer et al., 2012) was used to identify putative CNVs in the exome sequence of the 175 bulls. cn.MOPs is a multiple sample read depth CNV detection method that applies a Bayesian approach to decompose read variations across multiple samples into integer copy numbers and noise by its mixture components and Poisson distributions, respectively. cn.MOPS avoids read count biases along the chromosomes by modeling the depth of coverage across all samples at each genomic position. The exome version of the cn.MOPS program was run using the default parameters.

CNVs were then used to construct a set of copy number variable regions (CNVRs). A CNVR was constructed by merging CNVs across samples that exhibited at least 50% pairwise reciprocal overlap in their genomic coordinates. For example, suppose we have two CNVs, CNV1 beginning at position *a* and ending at position *b* and CNV2 running from *c* to *d* with *a* < *c* < *b* < *d*. If the reciprocal overlap between the two CNVs is at least 50% then they are merged into a CNVR which runs from *a* to *d* on the genome.

### Gene content and gene ontology

We identified genes from the Ensembl (Version 80; Cunningham et al., 2015) annotation of UMD 3.1 overlapping (both completely and partially) with detected CNVRs. Functions of protein-coding CNV genes were determined using the PANTHER classification system (Version 10.0, Mi et al., 2013).

Enrichment analysis of gene function was performed using PANTHER's implementation of the binomial test of overrepresentation. Significance of gene ontology (GO) terms was assessed using the default Ensembl *Bos taurus* GO annotation as the reference set for the enrichment analysis, and data was considered statistically significant at a Bonferroni corrected *P* < 0.05.

### Analysis of selective constraint in CNV genes

Pairs of orthologous genes between *Bos taurus* and *Homo sapiens* were identified using Biomart (Guberman et al., 2011). dN/dS ratios were then computed in MATLAB (2015) using the suggested protocol. Briefly, for each ortholog pair the nucleotide sequences were translated to amino acid sequences, which were then aligned using the BLOSUM50 scoring matrix. The gaps from the aligned amino acid sequences were then copied to their corresponding nucleotide sequences, producing a codon-aligned pair of nucleotide sequences. Lastly, the synonymous (dS) and nonsynonymous (dN) substitution rates of the codon-aligned sequences were computed using the *dnds* function. Pairs of input sequences that were too divergent, i.e., pairs exhibiting saturation of substitutions, were removed from further analysis because a sensible dN/dS ratio could not be computed. *P*-values from a one-tailed Wilcoxon rank-sum test were used to test the hypothesis that dN/dS ratios of cattle genes overlapped by CNV were significantly shifted toward higher values than those of non-overlapped genes, i.e., that selection pressure is relaxed for CNV genes.

### Tissue specificity analysis

Tissue specificity of genes overlapped by CNVRs was assessed using two types of expression data, microarray and RNA sequencing, encompassing 22 different tissues (Table 2). Raw data sets for experiments GSE41637, GSE55435, GSE71153, GSE73699, GSE73261, and GSE73159 were downloaded from NCBI's Gene Expression Omnibus (www.ncbi.nlm.nih.gov/geo), and the raw data for experiment ERP005899 was downloaded from EMBL-EBI's European Nucleotide Archive (http://www.ebi.ac.uk/ena).

**Table 2:** **Gene expression data sets**.

**Study**	**Tissue**	**Data type**	**Number of samples**
GSE73699	Mesenteric fat	Microarray	15
GSE73261	Spleen^*^	Microarray	16
GSE73159	Duodenum	Microarray	16
	Jejunum	Microarray	16
	Ileum	Microarray	16
GSE41637	Brain	RNAseq	3
	Colon	RNAseq	3
	Heart	RNAseq	3
	Kidney^*^	RNAseq	3
	Liver^*^	RNAseq	3
	Lung^*^	RNAseq	3
	Skeletal muscle	RNAseq	3
	Spleen^*^	RNAseq	3
	Testes	RNAseq	2
GSE55435	Hypothalamus^*^	RNAseq	8
	Pituitary gland	RNAseq	7
	Uterus	RNAseq	8
	Endometrium	RNAseq	6
	Ovary	RNAseq	8
	Subcataneous fat	RNAseq	8
	Liver^*^	RNAseq	8
	Longissimus dorsi muscle	RNAseq	8
GSE71153	Rumen	RNAseq	16
ERP005899	Adipose	RNAseq	7~14 pooled
	Duodenum^*^	RNAseq	7~14 pooled
	Hypothalamus^*^	RNAseq	7~14 pooled
	Kidney^*^	RNAseq	7~14 pooled
	Lung^*^	RNAseq	7~14 pooled

Tissues marked with ^*^were present in multiple studies.

The microarray data (experiments GSE73699, GSE73261, and GSE73159) was processed as follows. Individual CEL files were processed using the UPC function from the SCAN.UPC package in R (Piccolo et al., 2012, 2013). UPC is a quantitative approach for normalizing gene expression data that produces standardized expression values that estimate whether a gene is “active” in a given sample. The program outputs for each gene in a given sample a universal expression code (UPC), a number between 0 and 1 where larger values suggest a greater likelihood that the gene is expressed in the sample. The UPC function was run using the default parameters, and for each tissue a gene was considered to be expressed in the tissue if it had a UPC > 0.5 in at least one sample.

The RNA sequencing data (experiments GSE41637, GSE55435, GSE71153, and ERP005899) was processed as follows. Raw sequence reads in individual fastq files were first mapped to the UMD 3.1 genome assembly using Tophat (Version 2.0.1; Trapnell et al., 2009). The Cufflinks software (Version 2.2; Roberts et al., 2011) was then used to compute the fragments per kilobase of transcript per million mapped reads (FPKM) for paired-end reads and the analogous reads per kilobase of transcript per million mapped reads (RPKM) for single-end reads. Both software packages were run using the default parameters, and for each tissue a gene was considered expressed in the tissue if it had FPKM or RPKM > 1.0 in at least one sample. Note that some tissues, including duodenum, hypothalamus, kidney, liver, lung, and spleen, were included in two of the experiments. For these tissues, a gene was considered expressed if it passed the expression criterion in at least one of the two experiments. Genes belonging to both the set of expressed genes and our CNV gene set were classified as expressed CNV genes, while genes that were expressed but not overlapped by CNVs were classified as expressed neutral genes. The *P*-values from a one-tailed Wilcoxon rank-sum test were used to test the hypothesis that expressed CNV genes in cattle are expressed in fewer tissues than expressed neutral genes.

### Analysis of network centrality

Centrality of CNV overlapped genes in protein-protein interaction (PPI) networks was assessed using the *Bos taurus* interaction dataset from the STRING database (Franceschini et al., 2013). This dataset consisted of 3,904,694 interactions for 19,032 unique genes. Network centrality was measured by computing the degree of the representative node in the PPI network for each gene. *P*-values from a one-tailed Wilcoxon rank-sum test were used to test the hypothesis that node degrees of cattle genes overlapped by CNV were significantly shifted toward lower values than those of non-overlapped genes, i.e., that CNV genes are less central in PPI networks.

## Results and discussion

### CNVR discovery and statistics in the GPE bulls

Putative CNVs across the population of 175 bulls were identified using the exome cn.MOPS software package (Supplementary Table 1B). We chose to use the cn.MOPS package since it has been shown to have a lower false-positive rate than other exome CNV detection methods (Guo et al., 2013). CNVs were then merged across samples into CNVRs. In this work, we aimed to study common coding sequence CNVs across the *Bos taurus* genome. In an attempt to filter out possible false-positive and rare CNVs, CNVRs were filtered out if they were not present in at least 3 samples (>2% of the population). Note that the 2% threshold was chosen arbitrarily. A total of 74 CNVRs were filtered out in this step. The final set of CNVRs consisted of 57 CNVRs (48 on the autosomes and 9 on the X chromosome).

Sizes of the CNVRs ranged from 0.0018 to 1.56 Mb, with an average of 0.1419 Mb and a median of 0.0567 Mb. The CNVRs occupied a total of 5.27 unique Mb or 0.19% of the UMD 3.1 *Bos taurus* genome. Among the CNVRs, 30 showed copy number loss, 16 showed copy number gain, and 11 showed a mix of copy number loss and gain from different individuals. A full list of the CNVRs can be found in Supplementary Table 1A.

**Figure 1:**
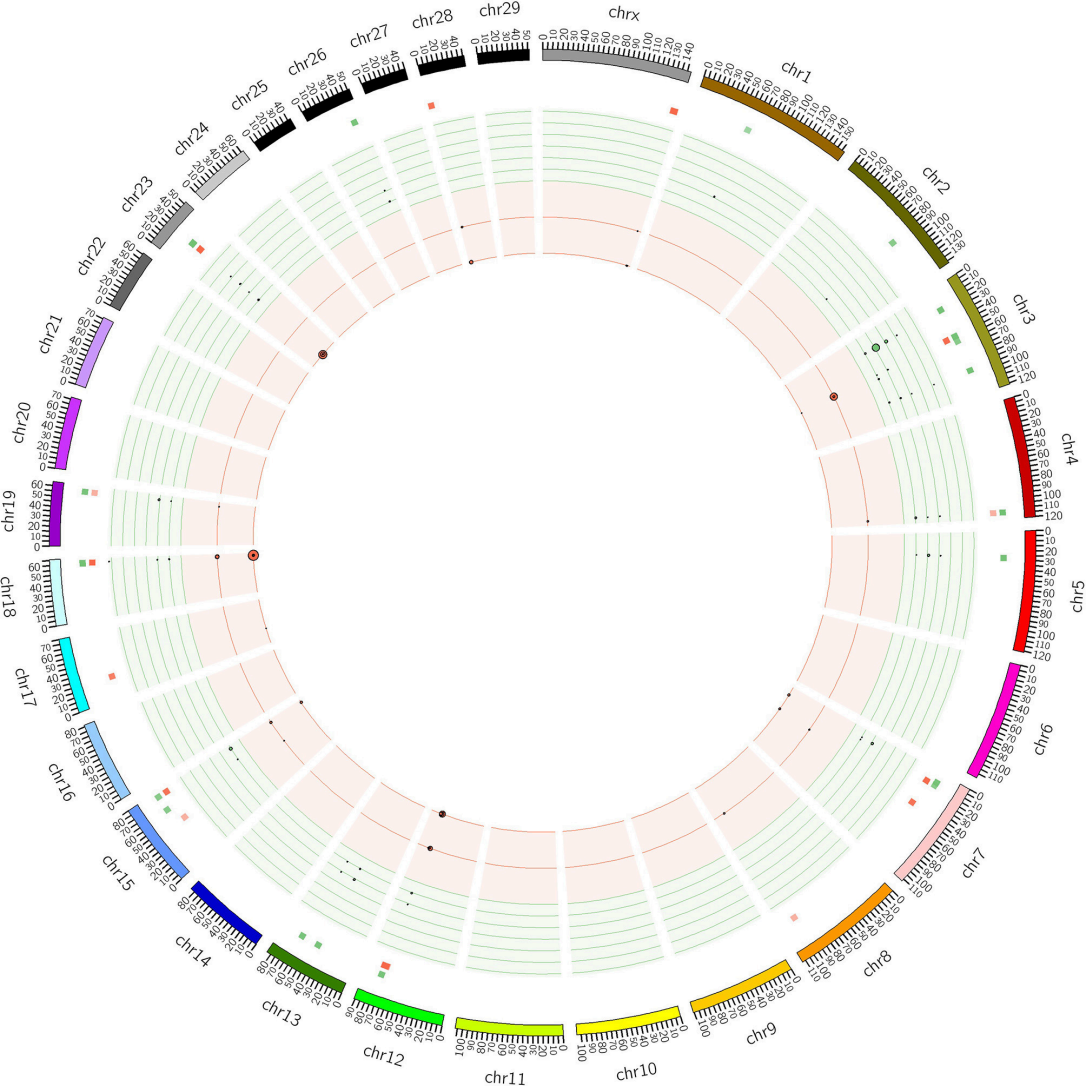
**CNVRs in GPE bulls**. Plot shows the CNVRs identified from the 175 sequenced GPE bull genomes in Circos format (Krzywinski et al., 2009). The outer ideogram runs clockwise from chromosome 1 to chromosome X with labels in Mb of physical distance. The copy number data is represented in the inner tracks. The two innermost tracks show scatter plots of the CNVRs, where the red track shows copy number loss and the green track shows copy number gain. The size of the dot in the scatter plot is proportional to the number of samples containing the CNVR. The other track shows a heat map which indicates the parts of the genome that contain copy number gain and loss. This plot simply collapses the scatter plot values onto a single radial position.

The distribution of CNVRs along each of the chromosomes is shown in Figure 1. Many CNVRs were present in a small number of bulls (24 of 57 were present in at most 5 bulls). One CNVR [CNVR 4 in Supplementary Table 1A] was present in 36% of the bulls. We observed some variation in the number of CNVRs between breeds. The greatest numbers of CNVRs were seen in Hereford (70), Angus (82), Simmental (72), and Red Angus (70), while the smallest numbers were seen in Braunveih (4) and Charolais × Angus (7). None of the CNVRs were breed-specific.

### Comparison of CNVRs with previous studies

Comparison of our results with autosomal CNVRs identified in several previous cattle studies showed varying levels of overlapping CNVRs between studies (Supplementary Table 2). In this analysis we used a much less stringent definition of overlapped CNVRs than in the rest of this work, where two CNVRs were considered overlapped as long as they shared at least one base. In order to compare some of the data sets to our results, we first had to map coordinates from the Btau 4.0 genome assembly to the UMD 3.1 assembly. This was done using the UCSC *liftover* tool (https://genome.ucsc.edu/util.html).

Array CGH with approximately 385,000 probes was used by Liu et al. (2010) to identify 200 CNVRs from 90 samples representing 11 different breeds, while Fadista et al. (2010) utilized the same technology with approximately 6.3 million probes to detect 254 CNVRs in 20 individuals from 4 breeds. The percentage of CNVRs from our results overlapping with these data sets was 18.8 and 70.8%, respectively.

A large variation in the number of detected CNVRs was seen in the SNP array-based studies. The number of CNVRs identified using the Illumina BovineSNP50 BeadChip ranged from 101 to 811. The two studies utilizing the Illumina BovineHD BeadChip had an even greater discrepancy in number of CNVRs, with 3438 CNVRs reported by Hou et al. (2012a) and only 247 CNVRs reported by Wu et al. (2015). The overlap of our results with these studies ranged from 0% in the BovineSNP50 chip studies of Hou et al. (2012b) and Jiang et al. (2012) to 79.2% in the BovineHD chip study of Hou et al. (2012a).

Comparing our results to other cattle CNVR sets generated from NGS we saw lower percentages of overlap. The study of Bickhart et al. (2012) identified 1265 CNVRs in the Btau 4.0 genome assembly. Their data consisted of WGS from 5 individuals representing 3 breeds, along with simulated NGS reads from the sequenced Hereford cow, L1 Dominette 01449. Only 2 of the CNVRs in our set overlapped with their data. Another NGS-based study, investigated copy number variation between one Holstein and one Black Angus bull (Stothard et al., 2011). A total of 790 CNVRs were identified in this study, and only 4 CNVRs from our set were found to be overlapping. In the NGS study of Zhan et al. (2011), 520 CNVRs were identified on the genome of one Holstein-Friesian bull when comparing the sequence reads against a Fleckvieh bull. A total of 7 of our CNVRs overlapped with this set. In a previous CNV study, we detected CNVRs from low coverage WGS of 154 pure bred bulls from 7 breeds used in the GPE project (Keel et al., 2016). The exome sequence of 117 of these bulls was used in the current study. Thirty one of our 57 CNVRs (64.6%) were overlapped by CNVRs from our previous study.

Generally speaking, percentages of overlap in CNV events identified between our study and previous studies were low, with an average of 30.9% of our CNVRs being overlapped by CNVRs in a previous study. This is similar to what we see when we compare previous studies (<40% overlap). These discrepancies are likely driven by many technical aspects, including vastly different sample sizes, differences in breeds and the number of breeds represented, detection platform (array-based vs. NGS), and CNV detection algorithms. The current study is one of the largest sequence-based cattle CNV studies to date, utilizing a larger sample size (175 samples) than previous NGS CNV studies, as well as samples from multiple breeds (10 breeds). It should be noted that the studies that had the highest percentage of overlap with the current study were those that had the largest numbers of breeds represented. This suggests that the inclusion of more breeds into CNV analyses may be crucial in identifying common CNVs across the *Bos taurus* genome and constructing a more comprehensive CNV map.

### Function of CNV genes

A total of 110 Ensembl genes from the UMD 3.1 assembly were identified to be CNV genes, overlapping (either completely or partially) with our detected CNVRs (Supplementary Table 4). These genes included 96 protein-coding genes, 7 snRNA, 6 pseudogenes, and 1 rRNA. Using PANTHER's functional annotation tool to inspect GO slim terms mapping to protein-coding CNV genes, we identified that many of these genes were involved in binding (35%), catalytic activity (23%), receptor activity (39%), signal transducer activity (36%), biological regulation (38%), cellular process (35%), and response to stimulus (48%).

Enrichment analysis was performed, using both the full *Bos taurus* GO database and the GO slim database, to identify GO terms that were significantly over- and underrepresented in our gene set. GO slim terms are a subset of the terms in the entire GO that give a broad overview of the ontology content. GO slim enrichment analysis showed that the terms extracellular transport, response to toxic substance, response to stimulus, response to interferon-gamma, amino acid transport, sensory perception of smell, G-protein coupled receptor signaling pathway, regulation of biological process, MHC protein complex, heterotrimeric G-protein complex, and plasma membrane were significantly overrepresented in the protein-coding genes overlapped by CNVRs (Bonferroni-corrected *P* < 0.05; Table 3). Results from the full GO database analysis are shown in Supplementary Table 3.

**Table 3:** **Significantly over- and underrepresented GO slim terms in the set of CNV genes**.

**Ontology Term**	**Gene Set (n genes)**				
	**Annotated genes^a^ (19879)**	**CNV genes^b^ (89)**	**CNV genes expected**	**Over (+) or Under (−)**	***P*-value**
**BIOLOGICAL PROCESS**					
Extracellular transport	53	6	0.24	+	4.16E-05
Response to toxic substance	45	5	0.20	+	5.08E-04
Response to stimulus	2880	43	12.89	+	9.08E-12
Response to interferon-gamma	58	4	0.26	+	3.50E-02
Amino acid transport	81	5	0.36	+	8.45E-03
Macrophage activation	131	6	0.59	+	7.18E-03
Sensory perception of smell	667	30	2.99	+	9.14E-20
Sensory perception of chemical stimulus	875	32	3.92	+	1.23E-18
Sensory perception	1108	32	4.96	+	1.19E-15
Neurological system process	1593	36	7.13	+	1.18E-14
System process	1809	36	8.10	+	6.23E-13
Single-multicellular organism process	2189	36	9.80	+	2.01E-10
Multicellular organismal process	2199	36	9.85	+	2.30E-10
G-protein coupled receptor signaling pathway	789	13	3.53	+	1.21E-02
Regulation of biological process	2260	34	10.12	+	1.36E-08
Biological regulation	2636	34	11.80	+	8.17E-07
Metabolic process	6613	14	29.61	+	3.88E-02
**MOLECULAR FUNCTION**					
n/a					
**CELLULAR COMPONENT**					
MHC protein complex	19	3	0.09	+	5.59E-03
Heterotrimeric G-protein complex	38	4	0.17	+	1.72E-03
Integral to membrane	1478	37	6.62	+	3.11E-17
Membrane	2433	37	10.89	+	2.19E-10
Plasma membrane	1458	24	6.53	+	1.01E-06
Cell part	4063	6	18.19	+	1.97E-02
Intracellular	3993	6	17.88	−	2.58E-02

a Number of genes in the background Bos taurus GO slim annotation set with given GO term. Total number of annotated genes is shown in parentheses.

b Number of CNV genes with given GO term. Total number of CNV genes with annotations in the background Bos taurus GO slim annotation set is shown in parentheses.

In addition, CNV genes were separated into three categories, duplication genes (genes overlapped by gain CNVs), deletion genes (genes overlapped by deletion CNVs), and mixed genes (genes overlapped by mixed CNVs) (Supplementary Table 3), and enrichment analysis was performed separately for each group. GO slim terms antigen processing and presentation of peptide or polysaccharide antigen via MHC class II, antigen processing and presentation, immune system process, and MHC protein complex were significantly overrepresented in the set of 25 genes overlapped by gain CNVs. For the 38 genes overlapped by deletion CNVs the terms response to toxic substance, response to stimulus, extracellular transport, sensory perception of smell, neurological system process, and regulation of biological process were significantly overrepresented. Genes overlapped by mixed CNVs had overrepresentation of GO terms response to interferon gamma, response to stimulus, response to toxic substance, sensory perception of smell, neurological system process, and regulation of biological process.

Several of the biological process categories identified for our cattle CNV have also been identified in other species. For example, MHC class II genes, olfactory receptors (OR), and amino acid transporters have been identified within CNV regions in humans (Schmidt et al., 2003; Traherne, 2008; Young et al., 2008). Human MHC class II and class III genes lie within CNVR in humans, and some of these have been linked to phenotypic variation like congenital hyperplasia, systemic lupus erythematosus disease risk, and host control of HIV-1 (Traherne, 2008). Olfactory receptors are G-protein coupled receptors involved in signal transduction. Young et al. (2008) showed that 18 OR and OR psuedogenes displayed varying copy numbers among 50 people. This variation may play a role in olfactory ability and sensitivity. Olfactory receptors may also play a chemosensory role as they are expressed on sperm and thought to direct them to the egg via chemotaxis (Spehr et al., 2006). Across several subspecies of the *Sus* genus, OR genes were also over-represented among CNVR (Paudel et al., 2015). These genes may have been important components of swine evolution, as scent would have been critical for foraging for food, avoiding predators, and finding a mate.

### Selective constraint on CNV genes

A central question in biology is how genomes evolve with respect to size and gene content and which factors affect and constrain this evolution. Intuitively, CNVs are likely to be subjected to selective pressure since large variants, in contrast with SNPs and other small variants, often affect entire protein-coding genes and substantial amounts of flanking DNA sequence.

It has long been hypothesized that gene duplications are drivers of both genome and gene function evolution. As described by Ohno (2013), when a gene duplication event first occurs, the two copies of the gene are assumed to be functionally redundant. It is believed that in most instances one copy of the gene will eventually be lost (pseudogenization or nonfunctionalization). However, as natural selection does not “know” which copy of the duplicated gene should be under selection and which should be free of selective constraint, both paralogs experience a period of relaxed selection. During this stage, it is possible that some divergence may be allowed and occasionally one copy may acquire a new function and subsequently be maintained by natural selection.

Rates of molecular evolution can be used to understand the selection constraints experienced by genes. In particular, contrasting the rate of protein-changing (non-synonymous) substitution and the rate of silent (synonymous) substitution at the nucleotide level allows us to identify the type of selection acting on individual genes. We measured selective constraint on cattle genes by using the dN/dS ratio. Here, dS denotes the synonymous substitution rate, and dN denotes the nonsynonymous substitution rate. When computed using sequences from divergent species, the dN/dS ratio is a measure of adaptive evolution in protein-coding sequences (Kryazhimskiy and Plotkin, 2008). For this reason we chose to use *Homo sapiens* as the comparison species since it is a well-studied organism, divergent from cattle.

Generally dN/dS ratios are interpreted as follows. dN/dS = 1 implies equal numbers of synonymous and nonsynonymous substitutions. This means that most variation is not caused by natural selection, but by random drift of mutant alleles that are neutral. dN/dS > 1 implies more nonsynonymous changes than synonymous. This means that there has been evolutionary pressure to escape the ancestral state, i.e., positive selection. Similarly, dN/dS < 1 implies a larger number of synonymous changes compared to nonsynonymous, meaning that there has been evolutionary pressure to conserve the ancestral state, i.e., negative selection.

dN/dS ratios were computed for orthologous pairs of genes (both CNV and neutral genes) between cattle and human (Supplementary Table 5). We first tested the hypothesis that, in general, compared to copy number neutral genes, CNV genes tend to be under relaxed selective pressure. This was done using a one-tailed Wilcoxon rank sum test, to test whether the median dN/dS ratio of all CNV genes was significantly higher that the median dN/dS ratio of neutral genes. We found that dN/dS ratios of CNV genes were significantly shifted toward higher values than neutral genes (Table 4), suggesting that CNV genes are subject to reduced selective constraint. This finding is consistent with previous results in both cattle and pigs (Fadista et al., 2010; Li et al., 2012).

**Table 4:** **dN/dS analysis**.

	**dN**	***P*-value**	**dS**	***P*-value**	**dN/dS**	***P*-value**
All CNV genes	0.1418	2.29E-09	0.5589	1.51E-07	0.2813	2.81E-06
Duplication genes	0.1601	1.45E-05	0.5135	0.0072	0.3151	3.01E-05
Deletion genes	0.1308	0.0142	0.5814	0.0083	0.2308	0.1068
Mixed genes	0.1235	1.36E-04	0.5681	4.79E-05	0.2702	0.0068
Neutral genes	0.0793	–	0.4288	–	0.1843	–

Median nonsynonymous (dN), synonymous (dS), and dN/dS rates are shown. P-values compare copy number variable genes with copy number neutral genes using a one-tailed Wilcoxon rank-sum test.

We also tested, individually, if duplication genes, deletion genes, and mixed genes tended to be under relaxed selective constraint compared to neutral genes. Both duplication and mixed genes were shown to have significantly higher dN/dS ratios than neutral genes, while dN/dS ratios of deletion genes did not differ significantly from those of neutral genes. The reduction in selective constraint observed in duplication and mixed genes follows Ohno's hypothesis that in a gene duplication event, one or both duplicates should experience relaxed selective constraint resulting in elevated rates of sequence evolution.

### Tissue specificity of CNV genes

Previous studies in fly (Dopman and Hartl, 2007) and mouse (Henrichsen et al., 2009) have shown that CNV genes tend to be more specific in their tissue expression patterns. We investigated this phenomenon in cattle using gene expression data from 22 different tissues (Table 2). Expressed CNV genes were expressed in fewer tissues (median = 2) than expressed neutral genes (median = 10) (one-tailed Wilcoxon rank-sum test, *P* < 0.00001). This is consistent with results from a similar study in fly (Dopman and Hartl, 2007), suggesting that CNVs occur more often in genes with tissue-specific expression than widely expressed genes that may have housekeeping functions.

A total of 6 CNV genes were identified to be tissue-specific in their expression (Table 5). Most of these genes (67%) were found in the testes. The most abundant gene family represented in this set, including 2 of the 4 genes, was the neuroblastoma breakpoint family (*NBPF*). Genes belonging to this family are involved in transporting RNA between the cell nucleus and the cytoplasm. *NBPF* genes have been shown to be copy number variable in humans and other primates (Vandepoele et al., 2005). This gene family has been shown to be expressed in the testes of humans (Vandepoele and van Roy, 2007) and is hypothesized to play a role in male reproduction (Vandepoele et al., 2005). The testis is a tissue that has a high level of interaction with the environment. Environmental factors, such as interference with testicular cooling and endocrine disruptors, are known to influence the development and function of the testes (Sharpe and Franks, 2002). Our finding tissue-specific CNV genes in the testes is perhaps not coincidental. It has been argued in previous studies that copy number variation is the result of positive selection for a diverse set of proteins that can meet the challenges of a constantly changing environment (Kondrashov and Kondrashov, 2006).

**Table 5:** **Number of tissue-specific genes with copy number variation**.

**Tissue**	**Number of tissue-specific genes**	**Number of tissue-specific CNV genes**
Testes	531	4
Brain	318	0
Spleen	81	1
Duodenum	6	0
Colon	40	1
Liver	69	0
Lung	45	0
Kidney	75	0
Ovary	0	0
Endometrium	0	0
Uterus	0	0
Rumen	117	0
Mesenteric fat	8	0
Adipose	0	0
Hypothalamus	0	0
Heart	14	0
Skeletal muscle	37	0
Pituitary gland	0	0
Subcutaneous fat	0	0
Longissimus dorsi muscle	0	0
Jejunum	6	0
Ileum	8	0

It should be noted that the tissues used in this analysis were downloaded from the NCBI database and did not originate from the same samples in which our CNV were detected. This is a major limitation in our tissue specificity analysis. As mentioned before, concordance between individual cattle CNV studies tends to be quite low. Cattle CNVs have also been shown to be lineage-differentiated (Xu et al., 2016). Therefore, it is quite possible that CNVs in the samples used for RNA sequencing could be quite different from those identified in this study. Hence, the tissue-specific expression patterns of CNV genes warrants further investigation using a dataset that includes whole-genome sequence as well RNA sequence from multiple tissues in the same set of samples.

### Network centrality of CNV genes

Protein centrality in PPI networks has been correlated with evolutionary rate and essentiality of genes in several species (Hahn and Kern, 2005). Proteins that are more central in PPI networks tend to evolve more slowly and be more essential. As shown above, CNV genes show a tendency to evolve more rapidly and are under reduced selective constraint. Therefore, it follows that the products of genes overlapped by CNV may be less central in PPI networks.

We tested this hypothesis using PPI data from the STRING database. We found that, in general, the number of interactors (i.e., the network node degree) for all CNV genes with ≥ 1 interaction was not significantly lower compared to neutral genes with ≥ 1 interaction (one-tailed Wilcoxon rank-sum test, *P* = 0.9137). Taking a closer look, we found that duplication genes did have significantly smaller numbers of interactors compared to neutral genes (one-tailed Wilcoxon rank-sum test, *P* = 0.0208), while deletion genes and mixed genes did not exhibit significantly lower numbers of interactors (*P* = 0.99 and *P* = 0.62, respectively). This finding is consistent with results in fly (Dopman and Hartl, 2007) and yeast (Li et al., 2006) in which products of duplicated genes were shown to have reduced network connectivity.

It is possible that a gene's copy number status may reveal information about its essentiality in PPI networks. The results above suggest that genes with lower network centrality may be more likely to have duplicates that are retained during evolution. We have shown that duplication genes are subject to reduced selective constraint, and as a result, they tend to undergo more rapid sequence evolution. Genes with high centrality in PPI networks may be more evolutionarily constrained since changes in protein coding could hinder the ability of the resulting protein to form interactions with other proteins in the network. Therefore, as hypothesized by Dopman and Hartl (2007), the set of genes with low numbers of interactions in PPI networks, populated by duplication genes in cattle, fly, and yeast, may experience reduced pleiotropy, and consequently be robust to structural mutations as well as less constrained during evolution.

## Conclusion

In recent years, copy number variation has gained considerable interest as a source of genetic variation that likely plays a role in phenotypic diversity. Much of the effort in studying copy number variation has been allocated to identification and validation of CNVs in several different organisms. Genome wide association studies have even linked changes in copy number to complex diseases. However, the evolutionary and functional impact of copy number variation is not well understood.

Cattle CNV research has made significant progress in the last 5 years. Genome-wide CNV maps have been generated using a variety of platforms and detection algorithms. However, the overlap between results from these studies is quite low. As mentioned earlier, these discrepancies may be due to differences in breeds, sample size, platform, and detection algorithm. In attempt to capture a larger portion of coding sequence copy number variation in the bovine genome, we chose to use a larger sample size (175 samples) than previous NGS CNV studies, as well as samples from multiple breeds (10 breeds). Additional copy number variation may be detected by including broader sampling from each from each breed and will likely be more effective in capturing breed-specific differences in CNV.

The evolutionary and functional patterns identified in this work for *Bos taurus* and in other studies for other species support a partial adaptive explanation for copy number diversity. We have shown that the dominant evolutionary forces that shape CNV are likely reduced functional (selective) constraint and mutational bias. Genomics research has traditionally concentrated on single-nucleotide polymorphisms as the most relevant source of structural variation in the genome. However, it is becoming progressively clear that CNVs may have considerable functional and evolutionary consequences. Understanding the role that CNVs play in reshaping gene structure, modulating gene expression, and ultimately contributing to phenotypic variation represent major future goals for the population genetics of structural variation.

## Author contributions

BK conceived of the study, and BK, AL-P, and WS participated in its design and coordination. WS mapped the exome sequence data, and BK performed all subsequent data analysis. BK drafted the manuscript, and all authors read and approved the final manuscript.

## Disclosure

Mention of trade names or commercial products in this publication is solely for the purpose of providing specific information and does not imply recommendation or endorsement by the U.S. Department of Agriculture. The U.S. Department of Agriculture (USDA) prohibits discrimination in all its programs and activities on the basis of race, color, national origin, age, disability, and where applicable, sex, marital status, familial status, parental status, religion, sexual orientation, genetic information, political beliefs, reprisal, or because all or part of an individual's income is derived from any public assistance program. (Not all prohibited bases apply to all programs.) Persons with disabilities who require alternative means for communication of program information (Braille, large print, audiotape, etc.) should contact USDA's TARGET Center at (202) 720–2600 (voice and TDD). To file a complaint of discrimination, write to USDA, Director, Office of Civil Rights, 1400 Independence Avenue, S. W., Washington, D. C. 20250-9410, or call (800) 795–3272 (voice) or (202) 720–6382 (TDD). USDA is an equal opportunity provider and employer.

### Conflict of interest statement

The authors declare that the research was conducted in the absence of any commercial or financial relationships that could be construed as a potential conflict of interest.
