# Supplementary material for: Evolutionary and Functional Features of Copy Number Variation in the Cattle Genome
Source: Front Genet. 2016 Nov 22;7:207. doi: 10.3389/fgene.2016.00207 (PMC5118444; doi:10.3389/fgene.2016.00207)
Supplement: Supplementary file 2 [file Table2.DOCX]

**Table S2.** Comparison of copy number variable regions on autosomes from this study and results from other studies (based on the UMD 3.1 bovine genome assembly).

|  | ***Findings from Other Studies*** | | | | ***CNVR Overlap With This Study*** | |
| --- | --- | --- | --- | --- | --- | --- |
| **Platform** | **Study** | **Breeds** | **Samples** | **# CNVRs**  **(# before mapping)** | **# CNVRs From This Study Overlapped** | **% CNVRs From This Study Overlapped** |
| CGH-based  study | Fadista et al. 2010 | 4 | 20 | 254 | 9 | 18.8% |
|  | Liu et al. 2010 | 17 | 90 | 200 | 34 | 70.8% |
| SNP-based  study (50K  chip) | Hou et al. 2011 | 21 | 521 | 743 | 33 | 68.8% |
|  | Bae et al. 2010 | 1 | 265 | 368 | 6 | 12.5% |
|  | Hou et al. 2012b^*^ | 1 | 472 | 500 (811) | 0 | 0% |
|  | Jiang et al. 2012^*^ | 1 | 2047 | 64 (101) | 0 | 0% |
|  | Sassi et al. 2016 | 1 | 1195 | 823 | 28 | 58.3% |
| SNP-based  study (HD chip) | Hou et al. 2012a | 27 | 674 | 3438 | 38 | 79.2% |
|  | Wu et al. 2015 | 1 | 792 | 247 | 14 | 29.2% |
|  | Aguilar et al. 2016 | 1 | 220 | 1662 | 32 | 66.7% |
|  | Prinsen et al. 2016 | 1 | 1410 | 563 | 33 | 68.8% |
|  | Xu et al. 2016 | 8 | 300 | 257 | 15 | 31.3% |
| Next-  Generation  Sequencing | Bickhart et al. 2012^*^ | 3 | 5 | 763 (1265) | 2 | 4.2% |
|  | Zhan et al. 2011^*^ | 1 | 1 | 419 (520) | 7 | 14.6% |
|  | Stothard et al. 2011^*^ | 2 | 2 | 634 (790) | 4 | 8.3% |
|  | Keel et al. 2016 | 7 | 154 | 1200 | 31 | 64.6% |
| This Study |  | 10 | 175 | 48 |  |  |

^*^ Original data set was mapped to BTAU 4.0 assembly. CNVRs were converted to UMD 3.1 coordinates using the UCSC *liftover* tool. Successfully mapped CNVRs are shown in the CNVR column with the original number of published CNVRs shown in parentheses.
