# Supplementary material for: Evolutionary and Functional Features of Copy Number Variation in the Cattle Genome
Source: Front Genet. 2016 Nov 22;7:207. doi: 10.3389/fgene.2016.00207 (PMC5118444; doi:10.3389/fgene.2016.00207)
Supplement: Supplementary file 3 [file Table3.DOCX]

**Table S2.** Significantly over- and underrepresented GO terms in the set of CNV genes.

|  | Gene Set (n genes) | |  |  |  |
| --- | --- | --- | --- | --- | --- |
|  |  | |  |  |  |
| **Ontology**  **Term** | Annotated genes^a^ (19879) | CNV genes^b^ (89) | CNV genes expected | Over (+)  or  Under (-) | P-value |
| **Biological Process** |  |  |  |  |  |
| arginine transmembrane transport | 17 | 5 | 0.08 | + | 1.25E-04 |
| amino acid transmembrane transport | 69 | 6 | 0.31 | + | 5.53E-03 |
| carboxylic acid transmembrane transport | 74 | 6 | 0.33 | + | 8.27E-03 |
| organic acid transmembrane transport | 77 | 6 | 0.34 | + | 1.04E-02 |
| amino acid transport | 82 | 6 | 0.37 | + | 1.49E-02 |
| L-lysine transmembrane transport | 17 | 5 | 0.08 | + | 1.25E-04 |
| L-alpha-amino acid transmembrane transport | 32 | 6 | 0.14 | + | 6.28E-05 |
| L-lysine transport | 17 | 5 | 0.08 | + | 1.25E-04 |
| L-ornithine transmembrane transport | 17 | 5 | 0.08 | + | 1.25E-04 |
| detection of chemical stimulus involved in sensory perception of smell | 943 | 34 | 4.22 | + | 2.24E-18 |
| detection of chemical stimulus involved in sensory perception | 975 | 34 | 4.37 | + | 6.35E-18 |
| sensory perception | 1282 | 35 | 5.74 | + | 3.16E-15 |
| neurological system process | 1523 | 35 | 6.82 | + | 6.64E-13 |
| system process | 1886 | 35 | 8.44 | + | 4.16E-10 |
| sensory perception of chemical stimulus | 1018 | 34 | 4.56 | + | 2.44E-17 |
| detection of chemical stimulus | 997 | 34 | 4.46 | + | 1.28E-17 |
| detection of stimulus | 1091 | 34 | 4.88 | + | 2.09E-16 |
| response to chemical | 3153 | 34 | 14.12 | + | 2.10E-03 |
| detection of stimulus involved in sensory perception | 1016 | 34 | 4.55 | + | 2.30E-17 |
| sensory perception of smell | 963 | 34 | 4.31 | + | 4.31E-18 |
| G-protein coupled receptor signaling pathway | 1597 | 34 | 7.15 | + | 2.08E-11 |
| macromolecule metabolic process | 5470 | 7 | 24.49 | - | 2.53E-02 |
| organic substance metabolic process | 6897 | 8 | 30.88 | - | 1.29E-04 |
| metabolic process | 7389 | 11 | 33.08 | - | 1.12E-03 |
| cellular metabolic process | 6330 | 8 | 28.34 | - | 2.13E-03 |
| primary metabolic process | 6497 | 8 | 29.09 | - | 9.52E-04 |
| developmental process | 3929 | 3 | 17.59 | - | 4.20E-02 |
| regulation of cellular macromolecule biosynthetic process | 3024 | 1 | 13.54 | - | 4.95E-02 |
| regulation of cellular biosynthetic process | 3228 | 1 | 14.45 | - | 1.80E-02 |
| regulation of biosynthetic process | 3270 | 1 | 14.64 | - | 1.46E-02 |
| regulation of metabolic process | 4882 | 3 | 21.86 | - | 3.89E-04 |
| regulation of cellular metabolic process | 4635 | 1 | 20.75 | - | 1.07E-05 |
| regulation of macromolecule biosynthetic process | 3121 | 1 | 13.97 | - | 3.07E-02 |
| regulation of macromolecule metabolic process | 4616 | 3 | 20.67 | - | 1.50E-03 |
| regulation of nucleobase-containing compound metabolic process | 3121 | 1 | 13.97 | - | 3.07E-02 |
| regulation of primary metabolic process | 4607 | 2 | 20.63 | - | 1.73E-04 |
| regulation of nitrogen compound metabolic process | 3324 | 1 | 14.88 | - | 1.11E-02 |
| negative regulation of cellular process | 3321 | 1 | 14.87 | - | 1.13E-02 |
| negative regulation of biological process | 3557 | 1 | 15.92 | - | 3.41E-03 |
| **Molecular Function** |  |  |  |  |  |
| L-ornithine transmembrane transporter activity | 17 | 5 | 0.08 | + | 3.98E-05 |
| L-amino acid transmembrane transporter activity | 55 | 6 | 0.25 | + | 4.76E-04 |
| amino acid transmembrane transporter activity | 85 | 6 | 0.38 | + | 5.83E-03 |
| carboxylic acid transmembrane transporter activity | 110 | 6 | 0.49 | + | 2.50E-02 |
| organic acid transmembrane transporter activity | 114 | 6 | 0.51 | + | 3.06E-02 |
| L-lysine transmembrane transporter activity | 18 | 5 | 0.08 | + | 5.27E-05 |
| basic amino acid transmembrane transporter activity | 22 | 5 | 0.10 | + | 1.42E-04 |
| arginine transmembrane transporter activity | 18 | 5 | 0.08 | + | 5.27E-05 |
| odorant binding | 232 | 19 | 1.04 | + | 2.18E-15 |
| antiporter activity | 77 | 6 | 0.34 | + | 3.31E-03 |
| active transmembrane transporter activity | 348 | 11 | 1.56 | + | 1.09E-03 |
| olfactory receptor activity | 943 | 34 | 4.22 | + | 7.13E-19 |
| transmembrane signaling receptor activity | 1663 | 34 | 7.45 | + | 2.17E-11 |
| transmembrane receptor activity | 1701 | 34 | 7.62 | + | 4.18E-11 |
| receptor activity | 1934 | 35 | 8.66 | + | 2.79E-10 |
| molecular transducer activity | 1934 | 35 | 8.66 | + | 2.79E-10 |
| signaling receptor activity | 1746 | 34 | 7.82 | + | 8.91E-11 |
| signal transducer activity | 1994 | 34 | 8.93 | + | 3.93E-09 |
| GTP binding | 355 | 11 | 1.59 | + | 1.33E-03 |
| guanyl ribonucleotide binding | 371 | 11 | 1.66 | + | 2.04E-03 |
| guanyl nucleotide binding | 372 | 11 | 1.67 | + | 2.09E-03 |
| G-protein coupled receptor activity | 1366 | 33 | 6.12 | + | 5.27E-13 |
| pyrophosphatase activity | 720 | 13 | 3.22 | + | 4.37E-02 |
| hydrolase activity, acting on acid anhydrides, in phosphorus-containing anhydrides | 723 | 13 | 3.24 | + | 4.56E-02 |
| hydrolase activity, acting on anhydrides | 729 | 13 | 3.26 | + | 4.97E-02 |
| protein binding | 5193 | 4 | 23.25 | - | 1.93E-04 |
| **Cellular Component** |  |  |  |  |  |
| plasma membrane | 4146 | 43 | 18.56 | + | 8.81E-06 |
| membrane | 8396 | 57 | 37.59 | + | 3.16E-02 |
| cell periphery | 4248 | 43 | 19.02 | + | 1.88E-05 |
| integral component of membrane | 5566 | 55 | 24.92 | + | 3.62E-08 |
| intrinsic component of membrane | 5630 | 55 | 25.21 | + | 5.86E-08 |
| membrane part | 6465 | 55 | 28.94 | + | 1.62E-05 |
| intracellular organelle part | 6085 | 8 | 27.24 | - | 1.10E-03 |
| intracellular part | 11174 | 13 | 50.03 | - | 5.72E-13 |
| intracellular | 11725 | 15 | 52.49 | - | 4.21E-13 |
| intracellular organelle | 9620 | 11 | 43.07 | - | 6.49E-10 |
| organelle | 10609 | 11 | 47.50 | - | 6.81E-13 |
| organelle part | 6222 | 8 | 27.86 | - | 5.82E-04 |
| nucleus | 5450 | 6 | 24.40 | - | 9.70E-04 |
| intracellular membrane-bounded organelle | 8602 | 8 | 38.51 | - | 1.29E-09 |
| membrane-bounded organelle | 9690 | 8 | 43.38 | - | 8.80E-13 |
| extracellular region | 3585 | 3 | 16.05 | - | 3.33E-02 |
| cytoplasmic part | 5586 | 3 | 25.01 | - | 1.50E-06 |
| cytoplasm | 8286 | 7 | 37.10 | - | 1.21E-09 |

^a^ Number of genes in the background *Bos taurus* annotation set with given GO term. Total number of annotated genes is shown in parentheses.

**^b^** Number of CNV genes with given GO term. Total number of CNV genes with annotations in the background *Bos taurus* annotation set is shown in parentheses.
