# Supplementary material for: Evolutionary and Functional Features of Copy Number Variation in the Cattle Genome
Source: Front Genet. 2016 Nov 22;7:207. doi: 10.3389/fgene.2016.00207 (PMC5118444; doi:10.3389/fgene.2016.00207)
Supplement: Supplementary file 6 [file DataSheet1.DOCX]

Supplementary Material

Evolutionary and functional features of copy number variation in the cattle genome

Brittney N. Keel^*^, Amanda K. Lindholm-Perry, Warren M. Snelling

*** Correspondence:**

Dr. Brittney Keel

U.S. Meat Animal Research Center

P.O. Box 166 Clay Center, NE 68933, USA
[brittney.keel@ars.usda.gov](mailto:brittney.keel@ars.usda.gov)

# Supplementary Tables

**Supplementary Table 1.** CNVRs (A) as well as putative CNVs (B) identified from whole exome sequence of 175 influential GPE bulls.

**Supplementary Table 2.** Comparison of copy number variable regions on autosomes between this study and previous studies.

**Supplementary Table 3.** GO term enrichment analysis for CNV genes.

**Supplementary Table 4.** Classification of CNV genes according to CNV type.

**Supplementary Table 5.** dN/dS ratios for orthologous pairs of genes in cattle and human.
